# Precision and accuracy of the subjective haptic vertical in the roll plane

**DOI:** 10.1186/1471-2202-11-83

**Published:** 2010-07-14

**Authors:** Jeanine R Schuler, Christopher J Bockisch, Dominik Straumann, Alexander A Tarnutzer

**Affiliations:** 1 Department of Neurology, Zurich University Hospital, Zurich, Switzerland; 2 Department of Otorhinolaryngology, Zurich University Hospital, Zurich, Switzerland; 3 Department of Ophthalmology, Zurich University Hospital, Zurich, Switzerland

## Abstract

**Background:**

When roll-tilted, the subjective visual vertical (SVV) deviates up to 40° from earth-vertical and trial-to-trial variability increases with head roll. Imperfections in the central processing of visual information were postulated to explain these roll-angle dependent errors. For experimental conditions devoid of visual input, e.g. adjustments of body posture or of an object along vertical in darkness, significantly smaller errors were noted. Whereas the accuracy of verticality adjustments seems to depend strongly on the paradigm, we hypothesize that the precision, i.e. the inverse of trial-to-trial variability, is less influenced by the experimental setup and mainly reflects properties of the otoliths. Here we measured the subjective haptic vertical (SHV) and compared findings with previously reported SVV data. Twelve healthy right-handed human subjects (handedness assessed based on subjects' verbal report) adjusted a rod with the right hand along perceived earth-vertical during static head roll-tilts (0-360°, steps of 20°).

**Results:**

SHV adjustments showed a tendency for clockwise rod rotations to deviate counter-clockwise and for counter-clockwise rod rotations to deviate clockwise, indicating hysteresis. Clockwise rod rotations resulted in counter-clockwise shifts of perceived earth-vertical up to -11.7° and an average counter-clockwise SHV shift over all roll angles of -3.3° (± 11.0°; ± 1 StdDev). Counter-clockwise rod rotations yielded peak SHV deviations in clockwise direction of 8.9° and an average clockwise SHV shift over all roll angles of 1.8° (± 11.1°). Trial-to-trial variability was minimal in upright position, increased with increasing roll (peaking around 120-140°) and decreased to intermediate values in upside-down orientation. Compared to SVV, SHV variability near upright and upside-down was non-significantly (p > 0.05) larger; both showed an m-shaped pattern of variability as a function of roll position.

**Conclusions:**

The reduction of adjustment errors by eliminating visual input supports the notion that deviations between perceived and actual earth-vertical in roll-tilted positions arise from central processing of visual information. The shared roll-tilt dependent modulation of trial-to-trial variability for both SVV and SHV, on the other hand, indicates that the perception of earth-verticality is dominated by the same sensory signal, i.e. the otolith signal, independent of whether the line/rod setting is under visual or tactile control.

## Background

Multimodal sensory input originating from vestibular (saccular and utricular macula, semi-circular canals) and extra-vestibular (truncal) graviceptors, skin and neck proprioceptors and vision is integrated by the central nervous system to generate an internal estimate of the direction of gravity (see [1] for review). The *subjective visual vertical *(SVV) is the most frequent method used to study how these different sensory systems contribute to graviception [2]. To measure the SVV, subjects are asked to adjust a luminous line in an otherwise dark environment along the perceived earth-vertical. Normative data, including the normal range of SVV deviations from earth-vertical, have been collected from healthy human subjects sitting in upright position [2,3] and in various whole-body roll-tilted positions [4-9]. Whereas in positions close to upright adjustments are accurate, systematic roll-angle dependent errors in SVV are known at larger whole-body roll tilts. Aubert [10] was the first to observe SVV undercompensation at whole-body roll angles larger than 60° ("A-effect"), peaking around 130° [5], while Müller [11] was the first to report the opposite phenomenon, i.e. SVV overcompensation, at roll angles smaller than 60° ("E-effect"). SVV overcompensation was later studied in more detail by others and was found to be either small or even absent [2,5-7,9,12]. At roll angles larger than 135° - 150°, SVV adjustments shift from undercompensation back to overcompensation, which might reflect a switch of the internal reference frame from pointing towards the head to pointing towards the feet [6,13]. Most likely, A- and E-effects are a consequence of how multiple sensory inputs are integrated into a unified percept of earth-vertical within the central nervous system [9,14].

Roll-tilting the head relative to gravity induces ocular counterroll (OCR). Although the position gain of OCR (= eye roll divided by head roll) is low in static conditions, being in the range of 5-25% of head-roll angle [15-21], orientation judgments that are made using visual indicators have been found to be somewhat affected by OCR [12]. Furthermore, rotating a visual line itself may induce ocular torsion in the same direction as the rotating line (ocular entrainment) and may thereby shift perceived visual vertical [22]. To avoid possible interference between visual orientation cues, OCR and verticality estimates, perceived vertical/horizontal has been studied in complete darkness using paradigms free from visual orientation cues, such as the subjective haptic vertical/horizontal (SHV/SHH) [12,23,24], verbal estimates of whole-body roll [5,25,26], or the subjective postural vertical/horizontal [9,27-29]. Among these paradigms, the SHV and the SHH are most closely related to the SVV and the subjective visual horizontal, respectively, as they also require indication of perceived orientation by aligning an object along the direction of estimated vertical/horizontal. In upright orientation, the SHV can be adjusted accurately within ± 3° [23]. Relative to the SVV, however, trial-to-trial variability of this haptic task was reported to be about twice as large [30].

Haptic perception results from the stimulation of mechanoreceptors in the skin, muscles, tendons and joints in the process of the manual exploration of an object in space [31,32]. To accurately perform a movement in the absence of visual guidance, both its starting point as well as the desired final position must be detected. Since proprioceptive sensors encode muscle lengths and joint angles, the initial frame of reference for haptic tasks is fixed to the limb segments. There is psychophysic evidence that this representation of joint angles is transformed to a space-fixed frame of reference (see [33] for review).

Studies measuring deviations in the SHV for whole-body roll angles up to 90° showed roll overcompensation up to ~12° [23,24] and head-on-trunk roll-tilts up to 35° showed roll overcompensation up to ~5° [34] or were accurate [35]. This suggests that estimates of vertical using distinct indicators (either a luminous line or a rod) may be based upon different sensory systems and/or may be processed within separate central networks. Such dissociations between the SVV and the SHV were reported also for lesions at the level of the vestibular nuclei. Whereas the SVV was severely tilted in upright position, the SHV deviated only marginally. This indicates that vestibular nuclear lesions may influence gravity perception by offsetting torsional eye position rather than by disrupting a single, internal representation of verticality [36].

Wade and Curthoys compared deviations from perceived earth-horizontal during eccentric constant-velocity rotation with chair velocities corresponding to roll-tilt angles of up to 40° during different psychophysical tasks (subjective visual horizontal or SVH; subjective haptic horizontal or SHH) and measured OCR using video-oculography [12]. They found the bimanually adjusted SHH to be free of constant errors, whereas the SVH deviated towards roll overcompensation. When subtracting the perceived earth-horizontal in the visually guided task from that in the non-visual haptic task, Wade and Curthoys noted a significant correlation between this difference and torsional eye position. Accordingly, they proposed that the brain is unaware of OCR and therefore systematic errors of perceived visual horizontal (or vertical) occur.

Unlike adjustments of the SVV, which resulted in errors of ~20° when positioned along the earth-horizontal axis, self-adjustments along the perceived horizontal axis in darkness are, on average, accurate [9,29]. Similarly, paradigms using verbal estimates of the actual whole-body roll angle (termed "subjective body tilt") instead of vision-based indicators yielded clearly smaller deviations of perceived earth-vertical [5,6,26] or were almost devoid of systematic errors [25].

These observations from various paradigms measuring the perceived earth-vertical (or horizontal) in complete darkness suggest that the underlying internal estimate of gravity is obtained by multisensory integration that is critically affected by the presence or absence of visual orientation cues. Whereas clear differences in the accuracy of the SVV and the SHV (and other paradigms related to estimating the direction of pull of gravity devoid of visual input) were demonstrated, similarities and differences concerning the precision of SVV and SHV adjustments under static conditions have not been addressed so far. Trial-to-trial variability of SVV increases with increasing whole-body roll [7,9,37], peaks around 120 to 150° roll [4,38,39] and reaches intermediate values in upside-down orientation, yielding an m-shaped modulation of SVV variability in the roll plane [8]. We previously suggested that this modulation is related to the properties of the otolith sensors (being non-uniformly distributed in the roll plane [40] and yielding a non-linear firing rate of the otolith afferents [41]) and to central computational mechanisms that are not optimally tuned for roll-angles distant from upright [8]. Whereas our simulations indicated a mostly central origin of the errors in SVV, the trial-to-trial variability of SVV adjustments was found to be only moderately altered by central mechanisms, which mainly led to an increase the middle-base of the "m" [8].

With SHV being most similar in how the task is performed compared to SVV, we aimed to focus on this non-visual, haptic paradigm when studying effects of visual orientation cues on internal estimates of earth-vertical. For internal estimates of verticality in non-visual paradigms, certain qualitative predictions about the accuracy and precision of adjustments can be made. Regarding the accuracy of the internal estimate of vertical, smaller adjustment errors are predicted for the SHV, since visual orientation cues are missing. This is based on both previous experimental findings in SHV for roll angles up to 90° [12,23,24] and modeling of SVV errors. SVV models based on optimal observer theory could successfully reproduce the pattern of adjustment errors by combining a noisy but accurate otolith signal with a bias signal [7,8,14,42] that points towards the head-longitudinal axis [43]. This strategy allows optimization of the precision of perceived earth-vertical and suggests that the adjustment errors emerge during central integration of this bias signal. Regarding the precision of internal estimates of vertical, we would expect a similar, m-shaped modulation in both SHV and SVV, based on the hypothesis that they share the same otolithic estimate of verticality independent of the presence/absence of visual orientation cues. This assumption underlines the eminent role of the otolith organs in obtaining a precise estimate of the direction of the gravity vector [39,44], which has recently received further support from a Bayesian model simulating the m-shaped pattern of SVV precision [8] and an analysis of the frame of reference of gravity estimates (reporting a mainly head-fixed reference frame [43]). An m-shaped modulation of SHV variability would therefore support the hypothesis that both paradigms rely on the same, mainly otolithic, input, whereas a modulation distinct from that observed in the SVV would suggest major differences in the sensors involved and in central processing when estimating earth-verticality without visual orientation cues.

In this study we assessed the accuracy and precision of the SHV in various whole-body roll positions. These findings were then compared with the previously reported pattern of SVV. In brief, we found that the direction of rod rotation significantly influenced SHV adjustment errors, indicating hysteresis. Whereas errors in the SHV paradigm were clearly smaller and showed a distinct pattern compared to the SVV paradigm, trial-to-trial variability was noted to modulate similarly in both SHV and SVV. These findings underline the important contribution of the central processing of visual input to errors in estimated earth-vertical and indicate that the precise perception of earth-verticality is dominated by the same sensory signal, i.e. the otolith signal, independent of whether the setting is under visual or haptic control. The hysteresis noted in the haptic modality underlines the importance of controlling for the direction of object rotation in future studies implementing the haptic vertical.

## Methods

### Subjects

Twelve healthy human subjects (5 women and 7 men; mean age (± 1 StdDev): 24.8 ± 2.9 years, age range: 21-30 years) were studied. Before enrollment handedness was assessed in all subjects according to their verbal report and only subjects that reported being right-handed were included. Subsequently, the 13-item questionnaire adapted by Chapman and Chapman [45] confirmed the handedness in eleven subjects (right-handedness defined as score in the range of 13 to 18 points; average score ± 1 StdDev of these 11 subjects: 14.5 ± 1.5), whereas one subject (HO) was identified as ambidextrous (score in the range of 19 to 32 points) with a score of 28 points by this assessment. Two participants were familiar with the experimental protocol. Informed consent of all subjects was obtained after full explanation of the experimental procedure. The experimental protocols were approved by the ethics committee at Zürich University Hospital and adhered to the Declaration of Helsinki for research involving human subjects.

### Experimental setting

Subjects were seated upright on a turntable with three servo-controlled motor driven axes (Acutronic, Jona, Switzerland). A thermoplastic mask (Sinmed BV, Reeuwijk, The Netherlands) that tightly covered the head in its neutral position (i.e. with the head- and body-longitudinal axis aligned) was applied and attached to the base plate behind the subject's head. Subjects were positioned so that the roll axis of the turntable intersected the center of the inter-aural line. A 4-point safety belt was placed around the shoulders and the hips. Pillows minimized movements of the trunk, the shoulders and the left arm; however, the right arm was spared to allow subjects to perform the rod adjustments without restraints. As the otolith organs, which have the largest impact on SVV [43], are situated in the head, the subject's orientation in the roll plane will be referred as *head-roll orientation*, although roll movements on the turntable were whole-body, i.e., included both head and trunk that were aligned. The rod to assess perceived earth-vertical was mounted on a safety bar in front of the subjects in the midline in 40 cm distance. This device consisted of a plastic tube, 29 cm long and 2.5 cm thick. By adding a Velcro strip to one end of the tube the two ends could be haptically distinguished. To achieve this haptic task, subjects actively explored the area in front of them in darkness with the right arm being unrestrained to determine the starting roll orientation of the rod. Subjects were allowed to reach the rod in a manner they felt most comfortable. Due to the fixed location of the rod 40 cm in the front of the midline, this will result in a slight forward movement of the unrestrained right arm, flexion in the elbow and an approximately horizontal position of the forearm for accurate grasping of the rod. Turntable position and rod orientation signals were digitized at 200 Hz and stored on a computer hard disk for offline processing.

### Experimental paradigm

**Figure 1:**
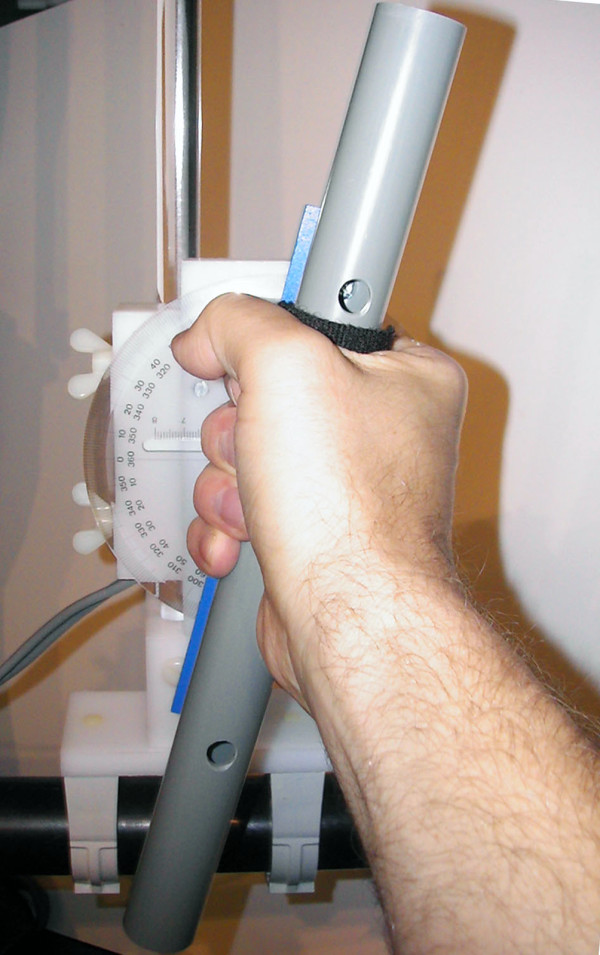
**Illustration of the wrap grip, showing a subject folding the fingers and the thumb of the dominant right hand about the rod**.

SHV adjustments were collected in 18 different head-roll positions to obtain a resolution of 20°. These positions were studied in pairs with a shift in roll position of 180° between trials (either in clockwise (CW) or counter-clockwise (CCW) direction). Changes in head-roll position were made in 8 s (turntable acceleration/deceleration: ± 10°/s^2^, peak velocity: ± 41°/s). Subjects were prompted acoustically to start the trial five seconds after the turntable stopped. They were asked to grasp the rod with their right hand using a wrap grip (Figure 1: the fingers and the thumb curl about the rod, see [46] for detailed taxonomy), align it to the perceived earth-vertical within 6 seconds by the shortest angle of rotation and to confirm the completion of the adjustment by pressing a button. This haptic task most closely resembles the paradigms previously used by Bauermeister [23] and Bortolami [24].

Before data collection, subjects practiced until trials could be performed within the time limit. This same time limit was used in all roll orientations to avoid possible influences of trial time on the variability of measurements. Whenever the confirm button was not pressed within 6 seconds, the trial was discarded and repeated later. The percentages of missed trials were below 5% in all subjects. We selected a short time interval since previous studies demonstrated that SVV trial-to-trial variability changes with head-roll position [4,9,37-39]. Thus subjects may experience more difficulties in setting the rod to earth-vertical at some (probably larger) roll angles than at others. Subjects could potentially compensate the roll-dependent imprecision by spending more time adjusting the rod to earth-vertical; consequently, comparisons of SHV variability at different roll angles would be hampered by unequal adjustment times. By setting the time limit short enough that subjects will spend about equal time for SHV adjustments in all roll positions such an effect can be minimized.

All trials were collected in complete darkness. To control for learning effects and possible effects of the direction of rod rotation, the order of starting rod positions was pseudo-randomized with random offsets between ± 38° and ± 82° relative to earth-vertical. By restricting the initial rod orientation to this range, subjects could grasp the rod comfortably without exceedingly large or difficult-to-achieve rotations of the wrist. A short break with the lights turned on was made at the end of each block, terminating dark adaptation and allowing the subjects to relax and remove the mask. At each roll position, 24 trials were collected, resulting in 432 trials for each subject recorded in three sessions (three blocks per session) on different days.

### Data analysis

Trials were sorted according to head-roll orientation and the direction of rod rotation. Outliers were defined as data points differing more than 2 standard deviations (StdDev) from the mean. 3.7% of trials were identified as outliers and discarded. Average deviations relative to earth-vertical and the StdDev were calculated for each subject. CW deviations relative to earth-vertical have a positive sign. We will use the term "trial-to-trial variability" whenever intra-individual StdDevs are reported. Analysis of variance (ANOVA, Minitab, Minitab Inc., State College, USA) with Tukey's correction for multiple comparisons was used for statistical analysis of the dependent variables (adjustment errors and trial-to-trial variability). Independent variables were the direction of rod rotation (CW vs. CCW), the head roll orientation and (when appropriate) the experimental paradigm (SHV vs. SVV). Both main effects of and interactions between independent variables were analyzed.

## Results

### Adjustment errors of the subjective haptic vertical (SHV)

**Figure 2:**
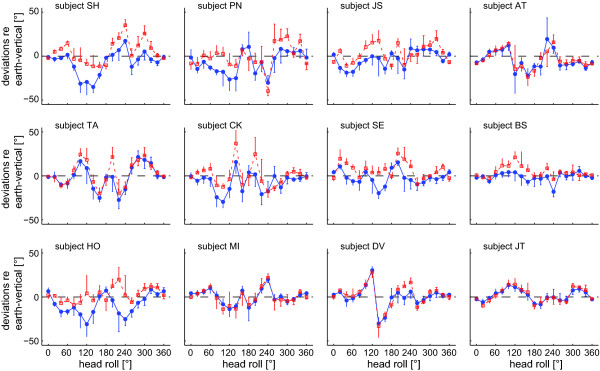
**Average (± 1 StdDev) deviations of subjective haptic vertical relative to earth-vertical are plotted against head roll, including data from all tested subjects**. Head-roll angles from 0 to 360° increase in CW direction. Blue circles: averages from trials with CW rotation. Red squares: averages from trials with CCW rod rotations. Dashed line: perfect earth-vertical rod adjustments.

Average individual SHV adjustments are shown for all subjects in Figure 2. A majority of subjects showed deviations of SHV adjustments at some roll angles (being most prominent in subjects TA, HO, and DV), however, no uniform pattern between subjects could be depicted and some subjects yielded minor deviations only (subjects SE, BS, MI, and JT). The direction of roll-angle dependent deviations varied considerably between subjects. For the SVV, sudden shifts from roll undercompensation to roll overcompensation in the range of 135 to 150° are known [6,13]. As a consequence of this shift, two separate clusters of SVV data points could be observed at certain roll angles. In the literature, this shift is considered to be a consequence of central processing (a shift in the reference frame used) rather than a consequence of the otolith sensors [8,13,26]. The SHV data was analyzed for such bistability, however, no systematic clustering of data points at given whole-body roll angles was observed.

**Figure 3:**
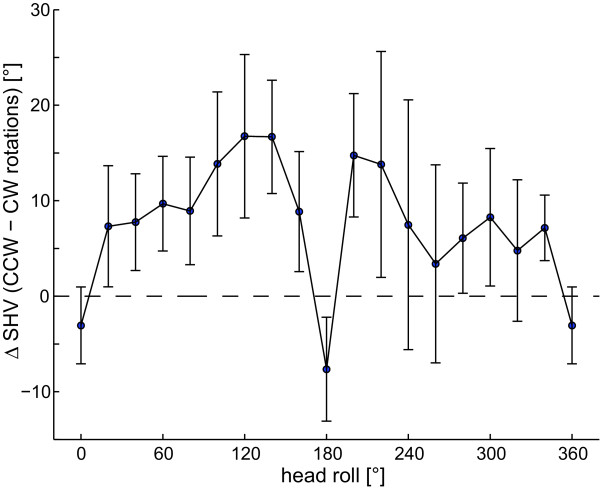
**Differences in SHV for CW and CCW rotations of the rod by the subject (Δ*SHV*)**. Overall average (± 1 StdDev) from trials with CW rod rotations were subtracted from trials with CCW rotations and plotted against head-roll angle. Positive values of Δ*SHV *reflect CCW shifts of trials with CW rod rotations relative to deviations of trials with CCW rotations.

**Figure 4:**
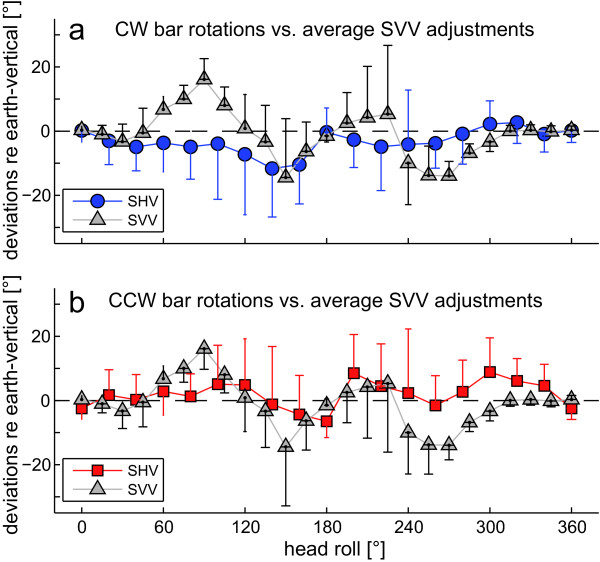
**Average (± 1 StdDev) deviations of SHV (blue and red circles) and SVV (gray triangles, from **[8]) **in all subjects relative as a function of head roll**. Note that trials with CW and CCW rotations of the SVV condition are pooled as no main effect for the direction of arrow rotation was previously noted [8]. Trials with CW rod rotations (blue circles) were shifted CCW relative to trials with CCW rod rotations (red squares) in most head-roll positions. Both for trials with CW rod rotations (upper panel) and for trials with CCW rod rotations (lower panel) the modulation of adjustments within the roll plane is clearly different from average SVV adjustments, showing deviations of smaller size and little roll-angle dependency.

As illustrated in Figure 3, averages of SHV adjustments obtained when subjects rotated the rod CW were shifted CCW relative to adjustments achieved by CCW rod rotations. Such direction of rotation dependent differences in adjusted SHV were noted in a majority of subjects; however, there was considerable inter-subject variability, as indicated by the error bars (± 1 StdDev). Rod roll direction-dependent differences increased with increasing head-roll angle. However, in upright and near upside-down orientation, they were inversed. We noted different average SHV errors depending on whether subjects were required to rotate the rod CW (Figure 4a) or CCW (Figure 4b). This pattern was already observed in a majority of individual subjects (see Figure 2). CW rod rotations resulted in average CCW shifts of perceived earth-vertical up to -11.7° at 140° head roll and an average CCW SHV shift over all roll angles of -3.3° (± 11.0°; ± 1 StdDev), and CCW rod rotations yielded peak SHV deviations in CW direction at 300° head roll of 8.9° and an average CW SHV shift over all roll angles of 1.8° (± 11.1°). Statistical analysis of SHV accuracy (2-way ANOVA with direction of rod rotation and head-roll orientation as independent variables) yielded a significant main effect for the direction of rod rotation (CW vs. CCW, F(1,22) = 27.4, p < 0.001) and for the head-roll orientation (F(17,204) = 2.14, p = 0.005), but showed no interaction between the two independent variables (F(17,204) = 1.03, p = 0.425), i.e. the effect of direction of rod rotation on haptic vertical adjustments was independent from the head-roll orientation.

**Figure 5:**
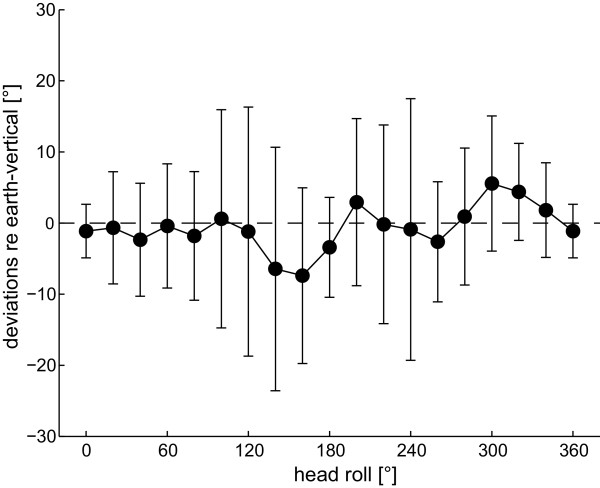
**Average (± 1 StdDev) deviations of SHV in all subjects as a function of head roll**. Trials with CW and CCW rod rotations are pooled. Dashed line: perfect earth-vertical rod adjustments.

For comparison, SVV values as reported by Tarnutzer et al. [8] using a similar paradigm are provided. As illustrated on Figure 4, SHV deviations were overall smaller and showed less modulation with roll angle. No shifts from roll overcompensation to roll undercompensation, as known in the SVV at large head roll angles [6], were observed in the SHV. Statistical analysis (3-way ANOVA with the experimental paradigm (SVV vs. SHV), direction of rotation of the device used to indicate vertical (CW vs. CCW), and head-roll orientation as independent variables) of those head-roll orientations studied in both paradigms (upright, 60° RED, 120° RED, upside-down, 120° LED, 60° LED), however, yielded no significant main effect for the paradigm (SVV vs. SHV, F(1,2) = 0.44, p = 0.508). Most likely these differences were not statistically significant due to their roll dependent modulations, resulting in large differences of adjustment errors between the two paradigms at some roll angles and in minor differences only at other angles. Furthermore, as only 6 head-roll orientations were studied in both paradigms this comparison does not reflect the modulation of SVV and SHV errors in the entire roll plane. When pooling trials with CW and CCW rod rotations (Figure 5) to allow comparison with previous studies of haptic vertical [23,24], we noted a tendency for both right-ear down and left-ear down roll-tilts towards roll overcompensation.

### Trial-to-trial variability in the subjective haptic vertical

**Figure 6:**
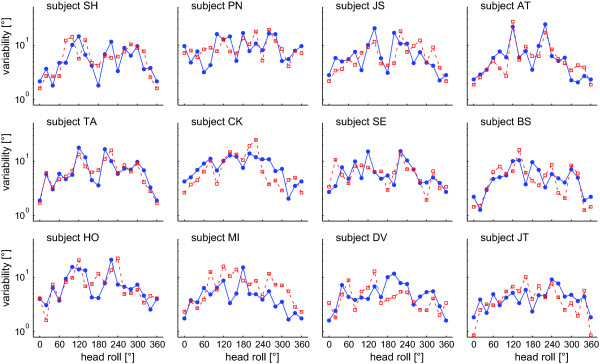
**Individual average (± 1 StdDev) trial-to-trial variability of SHV adjustments is plotted against the head-roll angle**. Note that the trial-to-trial variability is given in a logarithmic scale. Trials with CW (blue circles) and CCW (red squares) rod rotations are shown separately.

**Figure 7:**
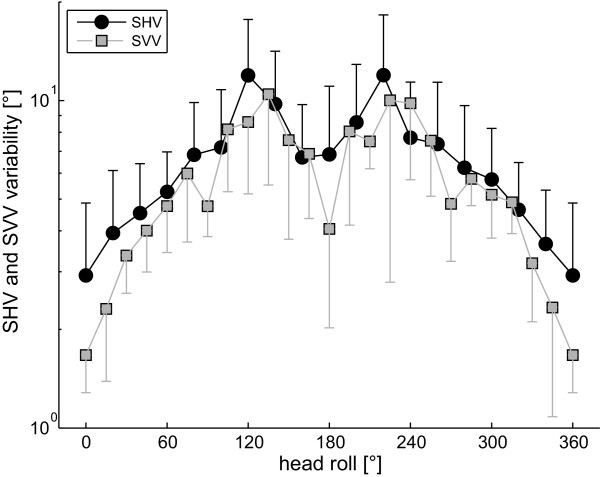
**Average (± 1 StdDev) trial-to-trial variability of SHV (black circles) and SVV (gray squares; from **[8]) **from all subjects is shown as a function of head roll**. Note that variability values are reported in a logarithmic scale. For both SVV and SHV, variability increased with increasing roll, peaked in the range of 120-150° and decreased again to intermediate values in upside-down orientation.

Individual trial-to-trial variability is shown in Figure 6. In all subjects, variability increased considerably with increasing roll angles, peaked around 120 to 140° head roll relative to upright and decreased again to reach a relative minimum near upside-down orientation. The difference between peak variability and the minima near upside-down varied between subjects. Since 2-way ANOVA yielded no significant differences between variabilities for CW and CCW rod rotations (F(1,22) = 0.24, p = 0.628), results were pooled for further analysis. The average trial-to-trial variability (Figure 7) shows a pattern of head-roll dependent modulation resembling an m-shaped curve, where the local minimum of the middle of the 'm' does not reach the base. Variability was minimal in upright orientation and increased with head-roll angle, peaking on average at 120 or 140° head roll. The minimum average trial-to-trial variability in upside-down was a factor of 2.3 larger than the variability in upright orientation, which is similar to the ratio between variability in upside-down and upright orientation previously reported for the SVV (2.4; [8]). Statistical analysis (3-way ANOVA; with the experimental paradigm (SVV vs. SHV), direction of rotation of the device used to indicate vertical (CW vs. CCW), and head-roll orientation as independent variables) comparing trial-to-trial variability at those head-roll angles studied in both the SVV (data taken from [8]) and the SHV paradigm (upright, 60° RED, 120° RED, upside-down, 120° LED, 60° LED), resulted in a main effect for the two paradigms (F(1,2) = 5.59, p = 0.019), indicating overall significantly larger trial-to-trial variability for the SHV. Furthermore, 3-way ANOVA indicated a significant interaction between the head-roll orientation and the type of paradigm (F(5,2) = 3.12, p = 0.01). As illustrated in Figure 7, variability (average ± 1StdDev) in or near upright position (2.9 ± 1.9° vs. 1.7 ± 0.4°; SHV vs. SVV in upright position) and in upside-down position (6.8 ± 4.2° vs. 4.1 ± 1.9°; SHV vs. SVV) was larger for the SHV than for the SVV and average differences diminished with intermediate-size head-roll angles. However, pairwise multiple comparisons (3-way ANOVA, Tukey corrected) yielded no significant differences (p > 0.05) between the SHV and the SVV variability for single head-roll angles.

## Discussion

When healthy human subjects were asked to indicate perceived earth-vertical in a haptic paradigm, the absence of visual orientation cues decreased the size of adjustment errors. The haptic task studied here, however, yielded a significant main effect for the direction of rotation of the rod, reflecting hysteresis, which was not the case for the SVV task used for comparison [8]. Overall, we noted a shift of perceived earth-vertical in CCW direction for CW rod rotations and a shift in CW direction for CCW rod rotations. These deviations varied little with changing head-roll orientation and were consistent with slight, non-significant undershooting of rod adjustments.

### Adjustments of the haptic vertical are subject to hysteresis

Whereas a distinct pattern of roll overcompensation (at small and very large head-roll angles) and roll undercompensation (at medium-sized roll angles) is known for the SVV [2,5,6], shifts in the SHV changed little with head roll and were independent from the direction of head roll-tilt (right-ear down vs. left-ear down). In pointing straight-ahead tasks and line bisection tasks used to indicate perceived body midline [47], similar deviations have been reported, showing overall slight leftward shifts and pointing from right-to-left ended significantly further right than pointing from left-to-right (see [48] for review). For bimanual kinaesthetic adjustments in a standing upright position, CW rotations yielded average adjustments shifted CCW relative to those obtained with CCW rotations [49,50]. The offsets noted for the unimanual haptic adjustments used here resemble this pattern. Such direction-dependent differences are referred to as hysteresis, i.e., indicate that the perceived position of the hand depends upon the recent history of hand positions. More specifically, hysteresis is a lagging or retardation of the effect, when the forces acting on a body are changed (Merriam Webster definition). Different mechanisms may yield hysteresis. For example, in visual processing short-term adaptation leads to changes in the orientation selectivity in the primary visual cortex [51,52], in vestibular stimulation the signal from the canals outlast the stimulation through velocity storage [53] and may bias subsequent vestibular-driven movements. In analogy, short-term adaptation could bias estimates of hand roll orientation towards the previously felt position of the hand. Short-term adaptation may therefore account for the finding that the SHV is not only determined by the internal estimate of the gravitational vertical, but depends also on the previous history of the hand position. Alternatively, hysteresis might arise from differences in the mechanics of the hand and the muscle groups involved, proposing that CW and CCW rotations of the rod held are not symmetric. Hogan studied the biomechanics of the upper extremities and demonstrated that the inertial resistance for hand movements is not uniform, but varies depending on the direction of hand movement [54]. Unit recording experiments indicate that this directional anisotropy is centrally compensated for when generating the motor output commands [55,56]. Possibly, the CNS fails to completely compensate for this directional anisotropy in the haptic alignment task used here, resulting in hysteresis when manually adjusting the rod.

Although the direction of arrow rotation in SVV paradigms is often not controlled for, hysteresis may emerge in certain conditions. Mezey et al. showed shifts in torsional eye position when watching at a slowly (4.8°/s) rotating visual line to obtain adjustments of SVV or subjective visual horizontal [22] into the direction of rotation. Even small changes of torsional eye position have previously been found to significantly change the perception of orientation [12,57]. The tilt-range where bistability of SVV adjustments (i.e., both A- and E-effects are present in single trials obtained at the same roll-tilt angle) occurred was found to depend on the direction of the preceding turntable rotation [13,58]. Thereby a short-path rotation (from upright by the shortest angle possible) was distinguished from a long-path rotation (from upright through the inverted position). This effect was associated with the subject's perceived body tilt signal, which was found to be accurate for short-path rotations and erroneous for long-path rotations [6]. In our study, changes in turntable position always resulted in rotations of 180° and the direction of preceding turntable rotation was varied pseudo-randomly to control for this kind of hysteresis. This property of our paradigms, however, did not dissolve hysteresis for the haptic alignments.

### How removing visual orientation cues affects errors in perceived vertical

To explain the systematic errors in the SVV, Mittelstaedt postulated an imbalance in the tilt signal due to an unequal number of hair cells in the two macular organs [9], based upon anatomical observations made by Rosenhall [59]. The proposed central compensational mechanism to minimize the effects of these imbalances, which integrates various sensory inputs into a unified percept of earth-vertical, is optimized for roll angles close to upright at the sacrifice of bigger errors at larger roll angles. If roll overcompensation and roll undercompensation were indeed consequences of the anatomical properties of the otolith organs, a similar pattern of roll-angle dependent modulations would be expected in any paradigm relying on otolith input. However, both the data obtained in the experiment presented here and findings in previous studies indicate that eliminating visual orientation cues reduces adjustment errors [9,12,29], although the otolith input remains unchanged. This, however, does not categorically exclude the possibility that the macular input is imbalanced as suggested by Mittelstaedt [9]. Alternatively the reduced adjustment errors when eliminating visual cues might be due to a distinct bias used in non-visual verticality tasks to compensate for imbalances in the otolith signal. Such a distinct bias signal for non-visual tasks could mask roll over- and undercompensation known from the SVV. However, such a bias signal has not been described yet. Also the brain might implement other mechanisms to reduce a bias originating from the otolith signal. Gravity acting on the outstretching hand could provide the CNS with an additional estimate of vertical. This information could be used to update a unified sense of the direction of gravity or to change the sensed body limb position. To overcome these uncertainties, more recent Bayesian modeling of both SVV errors [7,14,42] and SVV variability [8] focused on the precision of the roll-tilt signal. Bayesian frameworks have been used in the context of optimal observer theory when various sensory input signals are combined [60-63]. These Bayesian models make no assumptions about the utricular/saccular weighting and consider an accurate, but noisy otolith signal which is combined with prior knowledge about head roll to improve the estimate of earth-vertical. Prior knowledge drives the estimate of vertical towards the body-longitudinal axis as it is based on the assumption that small roll angles are most likely. Whereas noise reduction at small roll angles is thereby achieved, systematic errors at larger roll angles are caused [7,8].

### Possible explanations for the reduced adjustment errors when visual orientation cues are removed

Several mechanisms may explain the reduced adjustment errors when eliminating vision. Either prior knowledge - as it may be used in the SVV - is not implemented in non-visual verticality estimates. This would avoid roll over- and undercompensation at the sake of centrally optimizing the precision of verticality estimates through prior knowledge. Discarding prior knowledge in non-visual verticality estimates while implementing it in vision-based paradigms, however, seems rather unlikely, since in the SHV the trial-to-trial of adjustments was increased only slightly and non-significantly at larger head-roll angles compared to the SVV. This observation speaks for a central optimization of trial-to-trial variability for the SHV as well. Alternatively a distinct bias signal integrated in non-visual verticality tasks could have masked roll over- and undercompensation known from the SVV. The brain might implement other mechanisms to reduce the noise in the otolith signal as for example sense gravity acting on the outstretching hand. This information could potentially be used to update a unified sense of the direction of gravity or changes in the perceived body limb position. Increasing gravitational torques by adding loads to the forearm, however, did not improve the accuracy of forearm alignments with the vertical [64]. These observations speak against a significant contribution of sensed pull of gravity by the outstretched arm.

### Trial-to-trial variability of SVV and SHV show a similar roll-angle dependent modulation

Whereas the accuracy of perceived earth-vertical differed considerably depending on the pointing device used, the precision of verticality adjustments in the SHV followed a similar pattern as in the SVV. As a consequence, the m-shaped pattern of roll-angle dependent variability modulations described for the SVV [8] was reproduced when eliminating visual input and using a haptic alignment task instead. For the SVV an otolithic origin of the m-shaped variability modulation has been proposed [38,39]. This hypothesis received further support from an SVV model that was based on the otolith afferent properties (non-linear firing rate, non-uniform distribution) and central computational mechanisms that are not optimally tuned for roll-angles distant from upright. In these simulations the experimentally observed m-shaped pattern of variability could be reproduced [8]. In analogy to the parameters affecting SVV variability, we hypothesize that the m-shaped pattern in SHV variability is also of otolithic origin as these two tasks share similar sensory input (with the exception of visual input for the SHV and of haptic input for the SHV). However, there are notable differences in the m-shaped modulation of SHV variability compared to the SVV variability. Specifically, the middle part of the "m" is further away from the baseline and the overall trial-to-trial variability is greater. These differences could be related to the notion that central computational strategies also have a certain influence on the precision of verticality perception. We previously showed that the modulation of SVV precision could be reproduced significantly better in simulations when assuming efficient central computational mechanisms only near upright position [8]. Thereby differences in central computation strategies in SVV and SHV could lead to variations in the m-shaped pattern depending on the presence/absence of visual orientation cues.

As discussed above, systematic errors in the SHV adjustments were clearly smaller and distinct from the pattern know from the SVV. This raises the question to which extent central processing of the involved sensory signals becomes different when no visual input is available. In the simulations by Tarnutzer et al. it was shown that prior knowledge increases the precision of SVV adjustments at larger roll angles, whereas it was affected little near upright [8]. According to this model, the non-significantly increased SHV variability near upright compared to the SVV paradigm therefore seems not to be related to prior knowledge. More likely, the increased trial-to-trial variability near upright for haptic alignments is task-specific and may be related to distinct noise levels of the motor systems involved. Larger variability values for haptic tasks when compared to visual alignment tasks have also been reported by others [30].

### Comparison of SHV adjustments observed here with those reported in previous studies

Previously, Bauermeister et al. noted slight roll overcompensation (up to 6°) of unimanual right-handed SHV adjustments for head-roll angles up to ± 90° [23]. Bortolami et al. further characterized adjustments of the SHV in the range of ± 90° roll angles and noted roll overcompensation up to ~12° when roll-tilted left-ear down and accurate adjustments when roll-tilted right-ear down [24]. However, in both studies the direction of rod rotation was not controlled. When pooling trials with CW and CCW rod rotations in our study (Figure 5), we noted slight roll overcompensation both for right-ear down and left-ear down roll-tilts, which qualitatively resembles the findings reported by Bortolami et al. Deviations noted here were up to 6°, being in the range of deviations reported by Bauermeister et al. and smaller than the deviations noted by Bortolami et al. (up to 12°), however, inter-individual variability was considerable.

When using both hands to indicate perceived earth-horizontal, accurate adjustments were reported for roll-tilt angles up to 40° by Wade and Curthoys [12], whereas Bauermeister et al. noted deviations up to ± 5° of perceived earth-vertical for the same range of roll angles when using both hands [23]. When comparing results from the perceived horizontal with results from perceived vertical, however, caution is mandatory. For the SVV and the SVH, non-orthogonalities at large head-roll angles have been reported [25,65]. Similar non-orthogonalities have been reported for unimanual haptic adjustments [66] and for bimanual kinaesthetic adjustments [50]. Possibly, subjects had access to stronger haptic input in the paradigm by Wade and Curthoys or had more time to complete the adjustments. Alternatively, these differences might be related to the experimental stimuli. Whereas subjects were roll-tilted in the paradigm by Bauermeister et al., Wade and Curthoys simulated roll-tilts using centrifugation. Providing identical inter-aural shear to the otoliths but varying cranio-caudal shear by applying centrifugation or static head roll, centrifugation was found to yield larger ocular torsion (OT) [67], indicating relevant differences in how the otoliths are stimulated by these two experimental settings. Whether these discrepancies in stimulating the otolith afferents as reflected in OT affect psychophysical measurements as the SVV and the SHV as well, has not been studied, but proposes caution when comparing findings from centrifugation with static whole-body roll-tilts.

## Conclusions

Eliminating visual orientation cues improves the accuracy of internal estimates of the direction of gravity, whereas its precision is largely unaffected. These findings underline the important contribution of the central processing of visual input to errors in estimated earth-vertical and indicate that the precise perception of earth-verticality is dominated by the same sensory signal, i.e. the otolith signal, independent of whether the setting is under visual or haptic control. The significant direction-dependent differences in adjustment errors (hysteresis) noted in the haptic modality mandate the control for the direction of object rotation in future studies implementing the haptic vertical.

## Competing interests

The authors declare that they have no competing interests.

## Authors' contributions

JRS performed the experiments, participated in the design of the study and in drafting the manuscript. CJB participated in the study design and its coordination. DS helped formulating the study hypotheses and drafting the manuscript. AAT conceived of the study, performed the statistical analysis and participated in drafting the manuscript. All authors read and approved the final manuscript.

## References

[B1] Angelaki DE Gu Y DeAngelis GC Multisensory integration: psychophysics, neurophysiology, and computation Curr Opin Neurobiol 2009 19 4 452 458 10.1016/j.conb.2009.06.008 19616425 PMC2749464

[B2] Howard IP Human visual Orientation 1982 New York: Wiley

[B3] Friedmann G The judgement of the visual vertical and horizontal with peripheral and central vestibular lesions Brain 1970 93 2 313 328 10.1093/brain/93.2.313 5310320

[B4] Udo de Haes HA Stability of apparent vertical and ocular countertorsion as a function of lateral tilt Percept Psychophys 1970 8 3 137 142

[B5] Van Beuzekom AD Van Gisbergen JA Properties of the internal representation of gravity inferred from spatial-direction and body-tilt estimates J Neurophysiol 2000 84 1 11 27 10899179 10.1152/jn.2000.84.1.11/F

[B6] Kaptein RG Van Gisbergen JA Interpretation of a discontinuity in the sense of verticality at large body tilt J Neurophysiol 2004 91 5 2205 2214 10.1152/jn.00804.2003 14668294

[B7] De Vrijer M Medendorp WP Van Gisbergen JA Shared computational mechanism for tilt compensation accounts for biased verticality percepts in motion and pattern vision J Neurophysiol 2008 99 2 915 930 10.1152/jn.00921.2007 18094098

[B8] Tarnutzer AA Bockisch C Straumann D Olasagasti I Gravity dependence of subjective visual vertical variability J Neurophysiol 2009 102 3 1657 1671 10.1152/jn.00007.2008 19571203

[B9] Mittelstaedt H A new solution to the problem of the subjective vertical Naturwissenschaften 1983 70 6 272 281 10.1007/BF00404833 6877388

[B10] Aubert H Eine scheinbare bedeutende Drehung von Objekten bei Neigung des Kopfes nach rechts oder links Virchows Arch 1861 20 381 393 10.1007/BF02355256

[B11] Mueller GE Ueber das Aubertsche Phaenomenon Z Psychol Physiol Sinnesorg 1916 49 109 246

[B12] Wade SW Curthoys IS The effect of ocular torsional position on perception of the roll-tilt of visual stimuli Vision Res 1997 37 8 1071 1078 10.1016/S0042-6989(96)00252-0 9196725

[B13] Kaptein RG Van Gisbergen JA Nature of the transition between two modes of external space perception in tilted subjects J Neurophysiol 2005 93 6 3356 3369 10.1152/jn.01137.2004 15673551

[B14] De Vrijer M Medendorp WP Van Gisbergen JA Accuracy-precision trade-off in visual orientation constancy J Vis 2009 9 2 9 1 15 10.1167/9.2.9 19271919

[B15] Miller EF Graybiel A Counterrolling of the human eyes produced by head tilt with respect to gravity Acta Otolaryngol 1962 54 479 501 10.3109/00016486209126967 14473991

[B16] Diamond SG Markham CH Simpson NE Curthoys IS Binocular counterrolling in humans during dynamic rotation Acta Otolaryngol 1979 87 5-6 490 498 10.3109/00016487909126457 313656

[B17] Palla A Bockisch CJ Bergamin O Straumann D Dissociated hysteresis of static ocular counterroll in humans J Neurophysiol 2006 95 4 2222 2232 10.1152/jn.01014.2005 16338995

[B18] Ott D Seidman SH Leigh RJ The stability of human eye orientation during visual fixation Neurosci Lett 1992 142 2 183 186 10.1016/0304-3940(92)90369-I 1454213

[B19] Collewijn H van der SJ Ferman L Jansen TC Human ocular counterroll: assessment of static and dynamic properties from electromagnetic scleral coil recordings Exp Brain Res 1985 59 1 185 196 10.1007/BF00237678 4018196

[B20] Kingma H Stegeman P Vogels R Ocular torsion induced by static and dynamic visual stimulation and static whole body roll Eur Arch Otorhinolaryngol 1997 254 Suppl 1 S61 S63 10.1007/BF02439726 9065630

[B21] Bockisch CJ Haslwanter T Three-dimensional eye position during static roll and pitch in humans Vision Res 2001 41 16 2127 2137 10.1016/S0042-6989(01)00094-3 11403796

[B22] Mezey LE Curthoys IS Burgess AM Goonetilleke SC MacDougall HG Changes in ocular torsion position produced by a single visual line rotating around the line of sight--visual "entrainment" of ocular torsion Vision Res 2004 44 4 397 406 10.1016/j.visres.2003.09.026 14659966

[B23] Bauermeister M Werner H Wapner S The Effect of Body Tilt on Tactual-Kinesthetic Perception of Verticality Am J Psychol 1964 77 451 456 10.2307/1421016 14198668

[B24] Bortolami SB Pierobon A DiZio P Lackner JR Localization of the subjective vertical during roll, pitch, and recumbent yaw body tilt Exp Brain Res 2006 173 3 364 373 10.1007/s00221-006-0385-y 16628401

[B25] Van Beuzekom AD Medendorp WP Van Gisbergen JA The subjective vertical and the sense of self orientation during active body tilt Vision Res 2001 41 25-26 3229 3242 10.1016/S0042-6989(01)00144-4 11718769

[B26] Vingerhoets RA Medendorp WP Van Gisbergen JA Body-tilt and visual verticality perception during multiple cycles of roll rotation J Neurophysiol 2008 99 5 2264 2280 10.1152/jn.00704.2007 18337369

[B27] Anastasopoulos D Haslwanter T Bronstein A Fetter M Dichgans J Dissociation between the perception of body verticality and the visual vertical in acute peripheral vestibular disorder in humans Neurosci Lett 1997 233 2-3 151 153 10.1016/S0304-3940(97)00639-3 9350855

[B28] Bisdorff AR Wolsley CJ Anastasopoulos D Bronstein AM Gresty MA The perception of body verticality (subjective postural vertical) in peripheral and central vestibular disorders Brain 1996 119 Pt 5 1523 1534 10.1093/brain/119.5.1523 8931577

[B29] Mast F Jarchow T Perceived body position and the visual horizontal Brain Res Bull 1996 40 5-6 393 397 10.1016/0361-9230(96)00132-3 8886364

[B30] Kerkhoff G Multimodal spatial orientation deficits in left-sided visual neglect Neuropsychologia 1999 37 12 1387 1405 10.1016/S0028-3932(99)00031-7 10606013

[B31] Gibson JJ Observations on active touch Psychol Rev 1962 69 477 491 10.1037/h0046962 13947730

[B32] Revesz G System der optischen und haptischen Raumt228;uschungen Z Physiol 1934 131 296 375

[B33] Soechting JF Flanders M Moving in three-dimensional space: frames of reference, vectors, and coordinate systems Annu Rev Neurosci 1992 15 167 191 10.1146/annurev.ne.15.030192.001123 1575441

[B34] Guerraz M Luyat M Poquin D Ohlmann T The role of neck afferents in subjective orientation in the visual and tactile sensory modalities Acta Otolaryngol 2000 120 6 735 738 10.1080/000164800750000261 11099150

[B35] Funk J Finke K Muller HJ Utz KS Kerkhoff G Effects of lateral head inclination on multimodal spatial orientation judgments in neglect: Evidence for impaired spatial orientation constancy Neuropsychologia 2010 48 6 1616 27 10.1016/j.neuropsychologia.2010.01.029 20138897

[B36] Bronstein AM Perennou DA Guerraz M Playford D Rudge P Dissociation of visual and haptic vertical in two patients with vestibular nuclear lesions Neurology 2003 61 9 1260 1262 14610132 10.1212/01.wnl.0000086815.22816.dc

[B37] Dichgans J Diener HC Brandt T Optokinetic-graviceptive interaction in different head positions Acta Otolaryngol 1974 78 5-6 391 398 10.3109/00016487409126371 4451089

[B38] Lechner-Steinleitner S Interaction of labyrinthine and somatoreceptor inputs as determinants of the subjective vertical Psychol Res 1978 40 1 65 76 10.1007/BF00308464 635075

[B39] Schoene H Udo de Haes H Perception of gravity-vertical as a function of head and trunk position Zeitschrift für vergleichende Physiologie 1968 60 440 444 10.1007/BF00297938

[B40] Jaeger R Kondrachuk AV Haslwanter T The distribution of otolith polarization vectors in mammals: comparison between model predictions and single cell recordings Hear Res 2008 239 1-2 12 19 10.1016/j.heares.2008.01.004 18316166

[B41] Fernandez C Goldberg JM Physiology of peripheral neurons innervating otolith organs of the squirrel monkey. I. Response to static tilts and to long-duration centrifugal force J Neurophysiol 1976 39 5 970 984 824412 10.1152/jn.1976.39.5.970

[B42] Eggert T Der Einfluss orientierter Texturen auf die subjektive visuelle Vertikale und seine systemtheoretische Analyse PhD thesis 1998 Munich Technical Univ

[B43] Tarnutzer AA Bockisch CJ Straumann D Roll-dependent modulation of the subjective visual vertical: contributions of head- and trunk-based signals J Neurophysiol 2010 103 2 934 941 10.1152/jn.00407.2009 20018837

[B44] Miller EF Fregly AR Graybiel A Visual horizontal-perception in relation to otolith-function Am J Psychol 1968 81 4 488 496 10.2307/1421053 5760030

[B45] Chapman LJ Chapman JP The measurement of handedness Brain Cogn 1987 6 2 175 183 10.1016/0278-2626(87)90118-7 3593557

[B46] Cutkosky MR On Grasp Choice, Grasp Models, and the Design of Hands for Manufacturing IEEE Trans on Robotics and automation 1989 5 3 269 279 10.1109/70.34763

[B47] Jeannerod M Biguer B The directional coding of reaching movements. A visuomotor conception of visuospatial neglect 1987 Amsterdam: Elsevier

[B48] Jewell G McCourt ME Pseudoneglect: a review and meta-analysis of performance factors in line bisection tasks Neuropsychologia 2000 38 1 93 110 10.1016/S0028-3932(99)00045-7 10617294

[B49] Lejeune L Thouvarecq R Anderson DI Jouen F Kinesthetic estimation of the main orientations from the upright and supine positions Acta Psychol (Amst) 2004 117 1 13 28 10.1016/j.actpsy.2004.05.001 15288227

[B50] Lejeune L Thouvarecq R Anderson DJ Caston J Jouen F Kinaesthetic and visual perceptions of orientations Perception 2009 38 7 988 1001 10.1068/p6132 19764301

[B51] Dragoi V Sharma J Sur M Adaptation-induced plasticity of orientation tuning in adult visual cortex Neuron 2000 28 1 287 298 10.1016/S0896-6273(00)00103-3 11087001

[B52] Schummers J Sharma J Sur M Bottom-up and top-down dynamics in visual cortex Prog Brain Res 2005 149 65 81 full_text 16226577 10.1016/S0079-6123(05)49006-8

[B53] Raphan T Matsuo V Cohen B Velocity storage in the vestibulo-ocular reflex arc (VOR) Exp Brain Res 1979 35 2 229 248 10.1007/BF00236613 108122

[B54] Hogan N The mechanics of multi-joint posture and movement control Biol Cybern 1985 52 5 315 331 10.1007/BF00355754 4052499

[B55] Scott SH Sergio LE Kalaska JF Reaching movements with similar hand paths but different arm orientations. II. Activity of individual cells in dorsal premotor cortex and parietal area 5 J Neurophysiol 1997 78 5 2413 2426 9356393 10.1152/jn.1997.78.5.2413

[B56] Sergio LE Hamel-Paquet C Kalaska JF Motor cortex neural correlates of output kinematics and kinetics during isometric-force and arm-reaching tasks J Neurophysiol 2005 94 4 2353 2378 10.1152/jn.00989.2004 15888522

[B57] Pavlou M Wijnberg N Faldon ME Bronstein AM Effect of semicircular canal stimulation on the perception of the visual vertical J Neurophysiol 2003 90 2 622 630 10.1152/jn.00960.2002 12649316

[B58] Udo de Haes HA Schone H Interaction between statolith organs and semicircular canals on apparent vertical and nystagmus. Investigations on the effectiveness of the statolith organs Acta Otolaryngol 1970 69 1 25 31 10.3109/00016487009123333 5446607

[B59] Rosenhall U Vestibular macular mapping in man Ann Otol Rhinol Laryngol 1972 81 3 339 351 4113136 10.1177/000348947208100305

[B60] Knill DC Pouget A The Bayesian brain: the role of uncertainty in neural coding and computation Trends Neurosci 2004 27 12 712 719 10.1016/j.tins.2004.10.007 15541511

[B61] Kording KP Wolpert DM Bayesian integration in sensorimotor learning Nature 2004 427 6971 244 247 10.1038/nature02169 14724638

[B62] MacNeilage PR Banks MS Berger DR Bulthoff HH A Bayesian model of the disambiguation of gravitoinertial force by visual cues Exp Brain Res 2007 179 2 263 290 10.1007/s00221-006-0792-0 17136526

[B63] Laurens J Droulez J Bayesian processing of vestibular information Biol Cybern 2007 96 4 389 404 10.1007/s00422-006-0133-1 17146661

[B64] Darling WG Hondzinski JM Kinesthetic perceptions of earth- and body-fixed axes Exp Brain Res 1999 126 3 417 430 10.1007/s002210050748 10382626

[B65] Betts GA Curthoys IS Visually perceived vertical and visually perceived horizontal are not orthogonal Vision Res 1998 38 13 1989 1999 10.1016/S0042-6989(97)00401-X 9797945

[B66] Gentaz E Badan M Luyat M Touil N The manual haptic perception of orientations and the oblique effect in patients with left visuo-spatial neglect Neuroreport 2002 13 3 327 331 10.1097/00001756-200203040-00016 11930132

[B67] MacDougall HG Curthoys IS Betts GA Burgess AM Halmagyi GM Human ocular counterrolling during roll-tilt and centrifugation Ann N Y Acad Sci 1999 871 173 180 10.1111/j.1749-6632.1999.tb09183.x 10372070

